# Caregiver and Youth Characteristics That Influence Trust in Digital Health Platforms in Pediatric Care: Mixed Methods Study

**DOI:** 10.2196/53657

**Published:** 2024-10-28

**Authors:** Eric Chow, Alice Virani, Susan Pinkney, Fatema S Abdulhussein, Tibor van Rooij, Matthias Görges, Wyeth Wasserman, Jeffrey Bone, Holly Longstaff, Shazhan Amed

**Affiliations:** 1 Faculty of Medicine University of British Columbia Vancouver, BC Canada; 2 Department of Medical Genetics University of British Columbia Vancouver, BC Canada; 3 BC Children’s Hospital Research Institute Vancouver, BC Canada; 4 Department of Pediatrics BC Children’s Hospital Vancouver, BC Canada; 5 Department of Computer Science University of British Columbia Vancouver, BC Canada; 6 Department of Anesthesiology Pharmacology and Therapeutics University of British Columbia Vancouver, BC Canada; 7 Director Research Integration and Innovation Provincial Health Services Authority Vancouver, BC Canada

**Keywords:** pediatrics, patient trust, security, data privacy, data sharing, caregivers, patient engagement, co-design, personal health information, secondary use of data

## Abstract

**Background:**

Combining patient-generated health data and digital health platforms may improve patient experience and population health, mitigate rising health care costs, reduce clinician burnout, and enable health equity. However, lack of trust may be a notable barrier to the data-sharing required by such platforms. Understanding sociodemographic, health, and personal characteristics will enable developers and implementers of such technologies to consider these in their technical design requirements.

**Objective:**

This study aims to understand relationships between sociodemographic characteristics of caregivers of children or adolescents and trust in and willingness to use digital platforms to store and share personal health information for clinical care and research.

**Methods:**

This study used a mixed methods approach, including surveys of caregivers of youth aged <18 years living in Canada or the United States and youth aged 16 to 17 years living in Canada, as well as web-based bulletin board discussions to further explore topics of trust in data sharing. Sociodemographic and survey data were tabulated and explored using proportional odds ordinal regression models. Comments from web-based group discussions were analyzed thematically using a coding approach to identify issues important to the participants.

**Results:**

Survey data from 1128 caregivers (female participants: n=549, 48.7%; 36-50 years old: n=660, 58.5%; Canadian: n=603, 53.5%; urban population: n=494, 43.8%) were collected, of which 685 (60.7%) completed all questions. Data from 173 youth (female participants: n=73, 42.2%; urban population: n=94, 54.3%) were collected, of which 129 (74.6%) completed all questions, and data were available for analysis. Furthermore, among 40 participants, 23 (58%) caregivers contributed to the web-based discussion boards. Related to trust, living in a rural area (vs urban; odds ratio [OR] 0.66, 95% CI 0.46-0.95) resulted in lower concern for data privacy and security, while having an undergraduate (OR 1.82, 95% CI 1.30-2.55) or graduate degree (vs secondary or trade school; OR 2.50, 95% CI 1.68-3.73) resulted in higher levels of concern. Living with a chronic disease (OR 1.81, 95% CI 1.35-2.44) increased levels of concern regarding data privacy and security. Interestingly, those with chronic disease were more willing to use digital platforms for clinical care and share personal health information for not-for-profit research. Caregivers were most concerned about data breaches involving data from their children but also highlighted that digital platforms would allow for better coordination of care for their children.

**Conclusions:**

Our research confirms the willingness of caregivers and youth to use digital platforms for both clinical care delivery and research and suggests that the value of a digital platform may outweigh the risks of its use. Engagement of end users in co-designing such platforms has the potential to enhance digital trust. However, digital trust varies across sociodemographic groups; therefore, diverse end user engagement is necessary when designing digital applications.

## Introduction

### Background

Digital health can potentially advance the quintuple aim for health care improvement [1] by enhancing the patient experience, improving population health, mitigating rising health care costs, reducing clinician burnout, and enabling health equity [2,3]. Increased use of wearables that monitor health parameters, such as blood pressure, heart rate and rhythm, and interstitial glucose levels in real time produces vast amounts of patient-generated health data that, when combined with digital health platforms, can support remote patient monitoring, continuous (rather than episodic) care, and provide a more personalized care experience [4]. Moreover, patient-generated health and wellness data repositories provide research and quality improvement opportunities.

However, there are many obstacles to implementing digital health solutions [5]. Studies investigating the public’s perspective on sharing digital health data for clinical care and research have reported concerns related to trust in data sharing, such as lack of anonymity, vulnerability to cyberattacks, and fear of data breaches leading to data misuse [6,7]. Many authors have sought to define the criteria for trustworthiness in digital platforms [8-12] and have revealed key themes, including ease of use and ease of platform use, personal recommendations from other known users, and safety and privacy protection measures [8-14]. The reputation of digital providers and the quality of information are also perceived as fostering trust [12].

Trustworthiness, however, is influenced by a range of sociocultural and political factors [8,12], yet few studies have measured their magnitude of influence. In addition, the use of artificial intelligence in medicine is increasing [15]. However, patients have expressed concerns related to the possibility of misdiagnosis and privacy breaches [16], further highlighting the importance of understanding factors that promote trust in the design of digital health platforms.

As a mechanism to enhance the patient experience and improve population health, we are developing a digital health platform (TrustSphere) [17] for the secure sharing of patient-generated health data between patients and clinicians that enables a collaborative clinical care experience. This digital platform also provides opportunities for patients to share their patient-generated health data with researchers via a digitized consent process. Our first test use case is children living with type 1 diabetes (T1D), one of the most common childhood chronic diseases [18]. T1D is characterized by absolute insulin deficiency resulting in impaired blood glucose level regulation and serious lifelong complications, such as cardiovascular disease, kidney failure, and blindness. To mitigate the risk of these complications, individuals living with T1D (and their caregivers) must carefully monitor glucose levels 24 hours a day. Modern diabetes technologies, such as continuous glucose monitoring systems have been a “game changer” where, instead of using a glucose meter that requires finger pricks 4 to 6 times per day, patients wear a sensor that sits just under their skin and measures glucose levels every 1 to 5 minutes, with the data “pushed” to a smart device in real time [19]. Studies show that the use of continuous glucose monitoring systems compared with standard “finger prick” blood glucose monitoring resulted in significantly improved control of glucose levels in children and youth living with T1D [20]. However, the integration of these patient-generated glucose levels with digital health platforms that support a collaborative clinical care experience and provide opportunities for patients to participate in research is lacking, partly due to a lack of digital trust.

### This Study

We aimed to conduct a Canada and US-focused mixed methods study involving caregivers of children aged <18 years and youth aged 16 to 17 years to understand the relationship between sociodemographic characteristics (ie, sex, household income, level of education, rural vs urban locations, and experience with chronic disease) and “trust in” and “willingness to use” a digital platform to store and share personal health information (PHI) for clinical care and research. The United States and Canada are large North American countries with developed health care systems that are different yet share numerous similarities in their care models and associated challenges. Both nations grapple with escalating health care costs, inequitable access to care, and disparities in health outcomes. Moreover, there is a mounting level of concern in both countries regarding data security. We postulate that there will be differences in perspectives across different sociodemographic variables, and that understanding these differences will be important to consider in the design and prioritized features and functionalities of digital health platforms.

## Methods

### Study Population

Population groups that were approached for this survey study included caregivers of youth aged <18 years living in Canada or the United States (excluding Mexico) and youth aged 16 to 17 years living in Canada. Caregivers of children and youth living with T1D accessing care at the BC Children’s Hospital Diabetes Clinic (Vancouver, BC) were also invited to participate.

All survey respondents were offered the opportunity to participate in web-based bulletin board discussion groups that explored the topics of trust in data sharing. To be eligible for the web-based bulletin board discussion groups, participants had to be aged >18 years, living in Canada, have at least one healthy child or a child with a chronic disease who is aged <18 years, and be able to read, write, and understand English.

### Recruitment

Survey respondents were invited through the following methods: First, caregivers and youth living in Canada and the United States were invited by Insights West, a Canadian marketing research company that maintains a panel of volunteers to electively participate in web-based surveys and focus groups, along with their trusted panel partners (Dynata and Maru/Blue) from their list of adult volunteers. The youth included in this study were the children of the caregiver survey respondents living in Canada and were given parental consent to participate in this study. The target sample size was 1000; 1028 adult panel members and 173 youth responded. Caregivers of children living with T1D and receiving care at the BC Children’s Hospital Diabetes Clinic were also recruited via a clinical registry. The survey invitation was sent to 232 caregivers and 100 responded, resulting in a response rate of 43%. No financial incentives or honorariums were offered for survey participation.

The web-based bulletin board discussions were facilitated through 2 separate group discussions over 3 days in February 2021. A unique ID code identified caregiver participants who expressed interest in participating in the qualitative study on the quantitative survey; caregivers were not individually identifiable. Individuals who expressed interest were recontacted and asked additional screening questions (ie, age >18 years, living in Canada, have at least one child aged <18 years, and able to read, write, and understand English) before they were invited to participate. Invited caregiver participants then provided informed consent and received a link to the web-based bulletin board discussions. Participants in the bulletin board discussions were offered an honorarium of CAD $75 (US $57) in appreciation of their time.

### Data Collection

#### Overview

This mixed methods study was conducted from December 2020 to January 2021. The goal of the study was to gather caregiver and youth perspectives on elements of digital health delivery which included perspectives surrounding digital security, privacy and identity, ethics and informed consent, trust in digital health applications and platforms, sharing of digital health information, and perspectives on key features of an integrated digital platform for the delivery of clinical care and conduct of research.

#### Quantitative Methods

##### Survey Development

As no existing published validated surveys exploring these questions were available, the survey questions used in this study were collaboratively developed by the research team by drawing upon existing literature while also applying their expertise in qualitative and quantitative research methodologies, clinical care, patient engagement, privacy, procedural and substantive ethics and consent, digital health, and health informatics. The survey questions were also reviewed by 2 physicians in the Division of Endocrinology at BC Children’s Hospital and one health informatics researcher with expertise in questionnaire development to ensure clarity and relevance to current clinical practice. The survey underwent rigorous refinement; however, it was not pilot-tested. The following description of a digital platform was provided in the survey:

A secure online platform that will be customized for child and youth patients and their caregivers, and will integrate a patient’s health information such as diagnoses, medications and treatments, appointments, lab test results, wearable data (e.g. FitBit), etc. This platform would use secure and trusted digital identification, and follow the highest healthcare industry and public standards of privacy protection. The platform would help make it easier for children and families to access their health information and care plans, and to communicate directly with healthcare providers. It would also allow users to share their health information and care plans, if desired, with others involved in their child’s care, as well as donate their data confidentially for research.

The final survey for adults and youth comprised 32 to 36 questions (Multimedia Appendix 1) and 24 to 25 questions (Multimedia Appendix 2), respectively, depending on the responses and branching logic. The survey took 10 to 15 minutes to complete. The response options were predominantly Likert scales; however, some responses were binary, multiple selection, or rank order. There were no questions allowing for open-text responses. Respondents were able to skip questions, with no forced questions. All questions appeared on the screen except for branching questions that would only be displayed if relevant to the respondent’s prior answer.

##### Survey Dissemination

The main survey was provided to all caregivers, and a modified version of this survey was provided to youth, in which 10 caregiver-specific questions were removed. Insights West or its partners sent out invitations to participate via email. Invitations included a brief outline of the survey topic, the approximate time required to complete the survey, and a unique link to the web-based survey hosted by Insights West where each participant could submit a singular survey response. To protect anonymity, participant identifiers were kept separate from survey responses. The same survey as above was sent out by email by the clinic administrator to caregivers of children living with T1D and receiving care at BC Children’s Hospital Diabetes Clinic. No reminder emails were sent after the initial invitation.

#### Qualitative Methods

##### Discussion Guide Development

The qualitative discussion guide was codeveloped by Insights West in collaboration with the study team. The web-based bulletin board discussion group included 26 questions (Multimedia Appendix 3) about trust, data privacy, research, and whether families would use a digital health platform like TrustSphere. Of note, 23 of the original 26 discussion board questions were analyzed for this paper. The 3 discarded questions were unrelated to trust.

##### Discussion Board

Participants were asked to spend approximately 15 to 20 minutes per day answering questions over the course of 3 days. The total time was 45 to 60 minutes, and individuals could stop participating anytime. A moderator at Insights West monitored the discussion group daily, and follow-up questions were asked publicly to all participants or privately to specific participants as appropriate to probe for additional details. The moderator of the discussion board periodically met with the research team to review the discussion board and to guide moderation. The study team members could freely view the web-based bulletin board discussion and communicate with the moderator to guide probes. Transcripts of the written discussion questions, answers, and follow-up questions were recorded for qualitative analysis.

### Data Analysis

#### Statistical Analysis

Survey data were exported as an encrypted SPSS (IBM Corp) file and transferred to the research team through a secure file-sharing service. We used descriptive statistics to summarize respondent characteristics (both adult and youth) and to summarize responses to key questions around data storage, safety, trust, and use of a digital platform. The baseline characteristics (ie, age, gender, area of residence, level of education, and household income) of caregivers represented by respondents from Insights West and BC Children’s Hospital were similar, and therefore, caregiver data from both survey cohorts were amalgamated into a comprehensive “adult” category. The youth cohort was analyzed separately. The adult category was further subcategorized into adults with and without chronic disease and adults with and without a child with chronic disease. To assess the relationship between survey responses to key questions and priori selected sociodemographic variables, we used multivariable proportional odds logistic regression models. Missing data and responses for survey questions were recorded but were not included in statistical analysis. Results were summarized as odds ratios (ORs) and corresponding 95% CIs. Analyses were conducted using R (version 4.0.4; R Foundation for Statistical Computing) and Microsoft Excel.

#### Qualitative Analysis

Qualitative transcripts were transferred to the research team through secure file sharing. Data gathered from the 23 trust-related questions in the bulletin board discussions were analyzed using an inductive coding approach [21] to identify common themes. Initial codes identified by 2 investigators (AV and HL) were discussed, consolidated, and used to independently analyze all transcripts. All coded data were then systematically reviewed by AV and HL to ensure agreement, after which inductive analysis was used to generate themes and subthemes [21].

### Ethical Considerations

Ethics approval was obtained from the University of British Columbia/Children’s & Women’s Health Centre of British Columbia Research Ethics Board (approval number H20-03105, date of approval 2020-11-26, principal investigator: SA). Implied informed consent was used for surveys and discussion board participation. Findings were reported following the CROSS (Consensus-Based Checklist for Reporting of Survey Studies) checklist [20] for quantitative data and the SRQR (Standards for Reporting Qualitative Research) checklist [22] for reporting qualitative data for the bulletin board results as far as possible. The study data were deidentified with participant identifiers kept separate from survey responses. It is important to transparently state our team’s positionality. We come from diverse academic, cultural, and personal backgrounds, including different sex, races, ethnicities, and socioeconomic statuses. We acknowledge that our varying experiences and perspectives shape our approaches to methodology (ie, survey development) and data interpretation (ie, measures of socioeconomic status and comfort or trust with technology), and that privilege and bias may impact our work. We engaged in dialog and critical reflection to navigate these complexities ethically and responsibly to enhance the rigor of our research [23].

## Results

### Quantitative Survey: Perspectives on Digital Security, Sharing of PHI, and the Value of a Digital Platform

#### Overview

Out of 1128 caregivers, 685 (60.7%) responded to all questions, with a slightly greater number of caregivers being located in Canada (n=603, 53.5%) versus the United States (n=522, 46.2%). Among 173 youth, 129 (74.5%) responded to all questions. All the youth were from Canada. Among the 1128 caregivers, 231 (20.4%) reported being diagnosed with a chronic health condition, and 198 (17.6%) reported having a child diagnosed with a chronic condition. Among the 231 caregivers with a chronic health condition, 69 (29.9%) also had a child with a chronic health condition (Table 1).

**Table 1:** Demographic characteristics of study participants (n=1301).

Characteristics		Adults (n=1128), n (%)	Youth (n=173), n (%)
**Country of residence**			
	United States	522 (46.3)	—^a^
	Canada	603 (53.5)	173 (100)
	No response	3 (0.3)	—
**Sex**			
	Male participants	562 (49.8)	95 (54.9)
	Female participants	549 (48.7)	73 (42.2)
	Other	4 (0.4)	2 (1.2)
	Prefer not to answer	10 (0.9)	2 (1.2)
	No response	3 (0.3)	1 (0.6)
**Area^b^**			
	Urban	494 (43.8)	94 (54.3)
	Suburban	475 (42.1)	53 (30.6)
	Rural	137 (12.1)	22 (12.7)
	Do not know or prefer not to answer	12 (1.1)	1 (0.6)
	No response	10 (0.9)	3 (1.7)
**Level of education^c^**			
	Secondary school or trade school	168 (14.9)	—
	University or college (undergraduate degree)	669 (59.3)	—
	University or college (graduate degree)	267 (23.7)	—
	Prefer not to answer	16 (1.4)	—
	No response	8 (0.7)	—
**Household income^d^**			
	<CAD $75,000 (<US $57,000)	326 (28.9)	—
	CAD $75,000-$150,000 (US $57,000-US $114,000)	529 (46.9)	—
	>CAD $150,000 (>US $114,000)	216 (19.1)	—
	Prefer not to answer	55 (4.9)	—
	No response	2 (0.2)	—
**Age (y)**			
	18-35	143 (12.7)	—
	36-50	660 (58.5)	—
	51-65	250 (22.2)	—
	>65	52 (4.6)	—
	Prefer not to answer	3 (0.3)	—
	No response	20 (1.8)	—
**Parent with chronic disease^e^**			
	Yes	231 (20.5)	—
	No	766 (67.9)	—
	Prefer not to answer	25 (2.2)	—
	No response	106 (9.4)	—
**Child with chronic disease**			
	Yes	198 (17.6)	—
	No	873 (77.4)	—
	Prefer not to answer	21 (1.9)	—
	No response	36 (3.2)	—
**Families with children (y)**			
	<5	204 (19.8)	—
	5-7	209 (20.3)	—
	8-10	280 (27.2)	—
	11-13	306 (29.8)	—
	14-15	242 (23.5)	—
	16-17	240 (23.3)	—

^a^Data not collected.

^b^Area was self-defined by respondents as living in either an urban, suburban, or rural community.

^c^Level of education was defined as either the highest level completed or having some completion or in the process of completing.

^d^Household income was reported in the currency of the survey respondents’ country of residence; CAD $1=US $0.76.

^e^Chronic disease or illness was defined as medical conditions that lasted for a prolonged period and required ongoing medical care and lifestyle modifications to manage symptoms, prevent complications, and maintain one’s quality of life.

Table 2 shows the perspectives of caregivers and youth, stratified by caregivers with or without a chronic disease and caregivers with or without a child with a chronic disease, on: (1) knowledge about how and where PHI is stored and who has access to it; (2) trust in health care providers (HCPs), governments, and organizations (ie, hospitals) in keeping PHI secure; (3) willingness to share PHI for not-for-profit health research; and (4) the value and use of a digital platform as described in the survey (Methods section). Caregivers and youth had a similar understanding of where PHI is stored and who can access it, as well as similar trust that HCPs, governments, and organizations implement regulations to keep PHI secure. Compared with caregivers, more youth were willing to share their PHI for research and on a digital platform for clinical care. A larger proportion of caregivers with a chronic disease or a child with a chronic disease reported they understood who could access their child’s PHI compared with those without a child with a chronic disease. Further, among caregivers, having a child with a chronic disease resulted in greater trust that PHI is secure, more willingness to share data for research, and greater agreement that storing their child’s PHI on a digital platform would be a positive change.

When asked about their level of concern regarding web data privacy and security, almost all caregivers (1039/1128, 92.11%), youth (160/173, 92.5%), caregivers with a chronic disease (218/231, 94.4%), and caregivers of a child with a chronic disease (181/198, 91.4%) reported that they were *very* or *somewhat* concerned. Caregivers living with a chronic disease represented the respondent group with the highest level of concern, with 76.2% (176/231) reporting being *very concerned*. Among respondents’ selection of their top 3 choices of security processes that would make digital platforms more trustable, more than half of all caregivers indicated that features, such as multifactor authentication (539/1128, 47.78%) and notification of account changes and activity (504/1128, 44.68%) would improve their trust in a digital platform. These were closely followed by safety features such as using a trusted sign-in partner, for example, a banking or government services account (414/1128, 36.7%) and strong minimum password strength requirements (412/1128, 36.52%). The same 4 security features were most important for the youth (Multimedia Appendix 4).

**Table 2:** Perspectives of participants on aspects of data security and anticipated platform use and value (n=1301).

			All adults (agree; n=1128), n (%)		All youth (agree; n=173), n (%)		Adults with a chronic disease (agree; n=231), n (%)		Adults without a chronic disease (agree; n=766), n (%)		Caregivers of a child with a chronic disease (agree; n=198), n (%)		Caregivers of a child without a chronic disease (agree; n=873), n (%)
**Data storage**													
	I have a clear understanding of how and where my/my child’s PHI^a^ is stored.	765 (67.8)		125 (72.2)		180 (77.9)		512 (66.8)		134 (67.7)		592 (67.8)	
	I have a clear understanding of who can access my/my child’s PHI.	801 (71)		118 (68.2)		189 (81.8)		529 (69.1)		150 (75.8)		612 (70.1)	
**Trust**													
	I trust that my HCP^b^ will keep my/my child’s PHI secure.	976 (86.5)		145 (83.8)		203 (87.9)		654 (85.4)		184 (92.9)		750 (85.9)	
	I trust there are government regulations and practices in place to ensure that PHI is kept secure.	939 (83.2)		145 (83.8)		206 (89.2)		624 (81.5)		180 (90.9)		720 (82.5)	
	I trust there are organizational (eg, hospital) regulations and practices in place to ensure that PHI is kept secure.	969 (85.9)		144 (83.2)		208 (90.0)		642 (83.8)		188 (94.9)		736 (84.3)	
**Data sharing**													
	I am willing to share my/my child’s PHI if it creates progress in nonprofit health research.	816 (72.3)		137 (79.2)		184 (79.7)		523 (68.3)		176 (88.9)		603 (69.1)	
**Digital platform**													
	A platform like this would be a positive change in how my/my child’s PHI is stored.	790 (70)		138 (79.7)		171 (74.0)		515 (67.2)		169 (85.4)		585 (67.0)	
	I would share my/my child’s PHI on this platform with multiple providers.	690 (61.2)		122 (70.5)		159 (68.8)		435 (56.8)		156 (78.8)		505 (57.8)	
	I would find it overwhelming to have to keep track of another digital account.	687 (60.9)		126 (72.8)		163 (70.6)		459 (59.9)		122 (61.6)		529 (60.6)	

^a^PHI: personal health information.

^b^HCP: health care provider.

After being provided with a description of a digital platform (see above in Web-Based Survey Development), respondents were asked if they would be *likely*, *unlikely,* or *undecided* to use this platform. More youth (87/173, 50.3%) than caregivers (465/1128, 41.22%) stated they were *likely* to use the platform; however, a sizable portion of caregivers (407/1128, 36.08%) and youth (63/173, 36.4%) were *undecided*. A higher proportion of caregivers with a chronic condition (130/231, 56.3%) or who have a child with a chronic condition (130/198, 65.7%) responded that they were *likely* to use the described platform. Most caregivers were comfortable sharing their child’s PHI on a digital platform, including demographics (694/1128, 61.52%), contact information (639/1128, 56.64%), laboratory test results (770/1128, 68.26%), diagnoses (758/1128, 67.19%), medications, procedures and treatments (780/1128, 69.15%), medical imaging (777/1128, 68.88%), a list of their child’s HCPs (787/1128, 69.77%), health habits (such as physical activity and sleep habits; 788/1128, 69.85%), data from applications (751/1128, 66.58%), data from health devices (759/1128, 67.29%), infant feeding habits (760/1128, 67.38%), mental or emotional health (677/1128, 60.02%), immunization records (835/1128, 74.02%), family medical history (746/1128, 66.13%), dental health (850/1128, 75.35%), and allergies (838/1128, 74.29%). Overall, for all types of PHI, more caregivers with a chronic condition or caregivers of a child with a chronic condition were comfortable sharing their child’s PHI on a digital platform (Multimedia Appendix 5). Table 3 shows respondents’ perspectives on how helpful a digital platform would be for children and youth, caregivers, HCPs, and researchers.

Table 4 outlines the results of ordinal regression analysis for 3 key questions related to trust, willingness to share data for research, and the value of a digital platform.

**Table 3:** Perspective of participants on helpfulness of an integrated platform to different users (“How helpful do you think an integrated platform as described would be to/for”; n=1301).

Users	All adults (somewhat or very helpful; n=1128), n (%)	All youth (somewhat or very helpful; n=173), n (%)	Adults with chronic disease (somewhat or very helpful; n=231), n (%)	Adults without chronic disease (somewhat or very helpful; n=766), n (%)	Caregivers of a child with a chronic disease (somewhat or very helpful; n=198), n (%)	Caregivers of child without a chronic disease (somewhat or very helpful; n=873), n (%)
Children and youth	920 (81.5)	153 (88.4)	203 (87.9)	612 (79.9)	172 (86.9)	704 (80.6)
Parents and guardians	970 (86)	151 (87.3)	209 (90.5)	642 (83.8)	185 (93.4)	739 (84.7)
Doctors and other health care professionals	1023 (90.7)	162 (93.6)	215 (93.1)	689 (89.9)	193 (97.5)	789 (90.4)
Researchers	1013 (89.8)	154 (89.0)	210 (90.9)	681 (89.9)	192 (97.0)	774 (88.7)

**Table 4:** Ordinal regression analysisa: trust, willingness to share data, and likelihood of using the (described) digital platform.

		Q1: Level of concern around online data privacy^b^ (OR^c^<1 indicates lower level of concern; OR>1 indicates higher level of concern), odds ratio (95% CI)	Q2: Willing to share data for research^d^ (OR <1 indicates higher level of agreement; OR>1 indicates lower level of agreement), odds ratio (95% CI)	Q3: Likelihood of using a platform like this^e^ (OR<1 indicates more likely; OR>1 indicates less likely), odds ratio (95% CI)
**Age (y)**				
	18-35	1.18 (0.84-1.66)	0.85 (0.60-1.21)	0.63 (0.45-0.89)
	36-50	1.00 (reference)	1.00 (reference)	1.00 (reference)
	51-65	1.35 (1.03-1.77)	1.05 (0.79-1.39)	1.64 (1.25-2.17)
	>65	1.91 (1.11-3.33)	0.72 (0.41-1.29)	2.39 (1.39-4.10)
**Sex**				
	Male	1.00 (reference)	1.00 (reference)	1.00 (reference)
	Female	0.81 (0.65-1.03)	0.86 (0.68-1.09)	1.31 (1.05-1.65)
**Country**				
	Canada	1.00 (reference)	1.00 (reference)	1.00 (reference)
	United States	1.27 (1.00-1.60)	1.06 (0.83-1.35)	0.67 (0.53-0.85)
**Has a chronic disease**				
	Yes	1.81 (1.35-2.44)	0.71 (0.53-0.97)	0.63 (0.47-0.84)
	No	1.00 (reference)	1.00 (reference)	1.00 (reference)
**Child has a chronic disease**				
	Yes	1.42 (0.96-2.11)	0.51 (0.34-0.77)	0.34 (0.23-0.50)
	No	1.00 (reference)	1.00 (reference)	1.00 (reference)
**Area**				
	Urban	1.00 (reference)	1.00 (reference)	1.00 (reference)
	Suburban	0.72 (0.56-0.92)	1.32 (1.03-1.69)	1.57 (1.23-2.01)
	Rural	0.66 (0.46-0.95)	1.41 (0.97-2.04)	1.58 (1.11-2.26)
**Education**				
	Secondary or trade school	1.00 (reference)	1.00 (reference)	1.00 (reference)
	Undergraduate	1.82 (1.30-2.55)	0.88 (0.63-1.22)	1.11 (0.80-1.54)
	Graduate	2.50 (1.68-3.73)	0.93 (0.62-1.38)	1.03 (0.70-1.53)
**Income**				
	<CAD $75,000 (<US $57,000)	0.77 (0.58-1.02)	0.75 (0.57-1.00)	0.59 (0.45-0.78)
	CAD $75,000-$150,000 (US $57,000-US $114,000)	1.00 (reference)	1.00 (reference)	1.00 (reference)
	>CAD $150,000 (>US $114,000)	0.69 (0.48-0.98)	0.63 (0.44-0.92)	1.60 (0.42-0.85)

^a^Results of the ordinal regression are from a multivariate analysis. Each question included the same variables in their analysis.

^b^“In general, what is your level of concern regarding data privacy and security issues (eg, personal data being hacked, or companies such as Google or Facebook tracking your activities) when you are engaging in online activity?” (very unconcerned>very concerned).

^c^OR: odds ratio.

^d^“I’m willing to share some of my child’s/children’s health information confidentially if it helps create progress in nonprofit health research” (strongly agree>strongly disagree).

^e^“What is the likelihood that you would use a platform like this?” (very likely>very unlikely).

#### Trust

(Question: “In general, what is your level of concern regarding data privacy and security issues when you are engaging in online activity?”; OR<1=lower level of concern.) Caregivers living in suburban (OR 0.72, 95% CI 0.56-0.92) or rural areas (OR 0.66, 95% CI 0.46-0.95) were less likely to report a concern about web data privacy and security, compared with caregivers living in urban areas. In addition, those who completed an undergraduate degree (OR 1.82, 95% CI 1.3-2.55) and graduate degree (OR 2.5, 95% CI 1.68-3.73) compared with those who only completed secondary or trade school had higher odds of reporting concern regarding data privacy and security. Caregivers living with a chronic disease had higher odds of reporting concern (OR 1.81, 95% CI 1.35-2.44) than caregivers without a chronic disease.

#### Sharing Data

(Question: “I’m willing to share some of my child’s/children’s health information confidentially if it helps create progress in nonprofit health research.”; OR<1=higher level of willingness.) Compared with no chronic disease, caregivers living with a chronic disease (OR 0.71, 95% CI 0.53-0.97) or caring for a child with a chronic disease (OR 0.51, 95% CI 0.34-0.77) were more likely to be willing to share PHI for not-for-profit research.

#### Value of a Digital Platform

(Question: what is the likelihood that you would use a digital platform [as described in the survey]?; OR<1=more likely to use.) Compared with caregivers aged between 36 to 50 years, those aged between 18 to 35 years were more likely (OR 0.63, 95% CI 0.45-0.89), and those aged between 51 to 65 years (OR 1.64, 95% CI 1.25-2.17), and >65 years (OR 2.39, 95% CI 1.39-4.10) were less likely to use the described digital health platform. Compared with respondents located in Canada, those in the United States were more likely to use a digital platform (OR 0.67, 95% CI 0.53-0.85). Moreover, respondents living in suburban (OR 1.57, 95% CI 1.23-2.01) and rural (OR 1.58, 95% CI 1.11-2.26) areas were less likely to use a digital health platform when compared with those living in urban areas. Respondents living with a chronic health condition (OR 0.63, 95% CI 0.47-0.84) and those who have a child with a chronic condition (OR 0.34, 95% CI 0.23-0.5) were more likely to use such a platform.

### Qualitative Bulletin Board: Perspectives on Digital Security, Sharing of PHI, and the Value of a Digital Platform

Of the 40 caregivers who expressed interest in participating, 23 (58%) caregivers completed the web-based bulletin board discussion group process. Among the 23 participants, 11 (48%) were caregivers of a child with a chronic disease and 12 (52%) were caregivers with a healthy child.

The most common theme raised in the web-based discussions related to digital security concerns and the fear of a data breach being somehow connected to their child. One subtheme was participants’ worry that information they share about their child could be linked to their child in a way that might resurface in the future and impact job prospects or their ability to receive insurance. A second subtheme was parental concern that the information would be used for financial profit and be sold to third parties rather than be used altruistically. As stated during one web-based discussion:

[I am] not sure who would be really looking at this info...is it just the doctor that you’re dealing with...or can it be looked at by the receptionist that’s working at the office and gets ahold of your computer file and can get info on you?

Another participant stated, “I would worry about data being secure or sold to third parties.”

A second major theme was caregivers’ recognition of the benefits of digital health platforms. Caregivers were generally open to sharing information and appeared amenable to using the digital platform. Caregivers thought the digital platform would offer several benefits; subthemes of identified potential benefits included: (1) being able to access and share information more easily (test results); (2) saving time and effort coordinating their child’s health care; (3) being able to set or receive alerts and reminders for appointments, results, or action; (4) faster access to medical consultations or support (web-based or in person); (5) access to additional resources they might not be aware of; and (6) benefit to the child as they might be more comfortable interacting with the health care team through the digital platform rather than in-person.

As stated by one participant:

This is a great idea. The healthcare industry really needs to move into the 21st century. All people should have access to their own health records. As long as it is all secure, I am ok with it being online. I love the idea, especially in this COVID time, meeting my doctor virtually when it makes sense.

A third major theme was the trustworthiness of individuals and institutions involved with digital health platforms. One key subtheme was the importance of a trusted source of information when learning about the digital health platform. Web-based discussion board participants noted that they would prefer to be told about the digital platform by a trusted source (such as their physician or clinician) with the benefits, ease of use, and security information clearly outlined in any materials provided to them. When asked if they would seek out digital services like applications if their child were diagnosed with a chronic health condition, most stated they would talk to a clinician first as they place much trust in their physician. When asked about the digital platform described in the survey specifically, most reported that they would use it if it were recommended to them by their physician. Yet, if their physician did not recommend it, they might consider using it if other trusted sources (community groups, friends and family, the media) gave it positive feedback. They also might search on the web; however, a recommendation from a trusted source would make them more receptive to trying a new digital platform. When asked if their physician recommended this digital platform, one participant stated:

I would be more likely if they recommended it, yes. I would assume they have been educated on the positive aspects of the app and can see how this would benefit the child.

A second subtheme was trust in the recipients with whom they might share their health data. When asked with whom they were most comfortable sharing their child’s PHI, most cited their family physician or other health professionals. Respondents were also willing to share PHI with hospitals, Canadian research institutions, universities, specialists, their child’s school, and government agencies (eg, Health Canada).

The fourth theme generated in the web-based discussions was the tension between the barriers and benefits of additional new digital accounts and technology. Over half of the web-based discussion group participants (13/23, 57%) reported that they would find it overwhelming to keep track of another digital account. Some were concerned that there might be a steep learning curve or that using the platform might be challenging. A few participants explained, “I don’t need any more technology in my life.”

However, other participants disagreed and stated that once they were over the initial learning curve, adopting this digital platform would allow their child to receive better care coordination, making it worthwhile. They explained that it was easy for the password to be saved on their phone, so they were not concerned about additional tasks required by the digital platform. As explained by one participant:

I have a lot of digital accounts, I feel it is the way of the world. I don’t think one more would be an issue. Plus, there is a lot of information here, I feel like this is a one-stop shop for all our healthcare-related info. If I had to have multiple healthcare accounts, I would find it overwhelming, but since everything health-related seems to be in one spot, it is quite handy.

## Discussion

### Principal Findings

We identified novel associations between sociodemographic factors and trust in digital health applications to share PHI for clinical care delivery and research among caregivers of children and youth aged 16 to 17 years. We found that living in an urban area (vs rural), having an undergraduate or graduate degree (vs secondary or trade school), and having chronic disease experience (vs no chronic disease experience) increased the level of concern regarding data privacy and security. Interestingly, those with chronic disease experience had the highest level of concern yet compared with those without chronic disease experience, were more willing to share PHI for not-for-profit research and were more likely to use a digital platform for clinical care and chronic disease management. Studies have mostly reported on the perspectives of adults’ willingness to share personal data for research [6]. Our study adds to this growing literature by reporting on the perspectives of caregivers as delegates of their children on digital trust and digital platform use as it relates to sharing PHI for clinical care delivery and research.

### Comparison to Previous Work

Lack of digital trust has notably hindered the widespread adoption of digital health platforms [6,24]. Health care is increasingly being delivered in digital and virtual environments, making strong digital trust and identity necessary to support 2-way interactions between clinicians and patients, and patient participation in research. To unlock the potential of digital health, it is critical to understand the differing perspectives on digital trust of citizens across different sociodemographic groups when designing digital health applications to optimize usability, feasibility, and adoption [25]. Like our study, other studies assessing perceptions of digital services, such as the internet or social media, have demonstrated that individuals with lower levels of education tended to be less concerned with web privacy [26,27], while those with a college or graduate degree were more likely to take additional security measures, such as encrypting emails to protect their privacy [26]. Age also influences the adoption of digital health technologies [28,29] and people’s willingness to share personal health data for research, although our study did not show this. In a survey study, Nunes Vilaza et al [6] found that, compared with older individuals (>27 years), younger participants (<27 years) were more willing to share personal data for health research.

We observed that caregivers with chronic disease experience had the highest level of concern for data privacy and security yet were more likely to use a platform like TrustSphere for clinical care and share personal health data for not-for-profit research. Nunes Vilaza et al [6] found no difference between individuals who self-reported having good, very good, or excellent health compared with those who self-reported fair or poor health in their willingness to share PHI for research. Robbins et al [30] examined health application use stratified by self-reported health and chronic disease status. They found that individuals who self-reported very good or excellent health, compared with poor health, were likelier to download health applications. However, no significant difference in downloading health applications was found when examined based on chronic disease status. The results of our study show that trust in digital health may be connected to one’s familiarity with the health care system and the challenges that patients face in accessing their PHI.

Studies show that individuals with chronic illness are more likely to make altruistic choices, including participating in clinical research [31,32]. In addition, individuals with chronic disease experiences may view the loss of privacy as worthwhile to progress medical research and to benefit others or future patients [6,33]. Involvement in clinical research and the use of digital health services is often an empowering experience for participants as many gain additional connections to health care professionals or other similar individuals through their participation or access relevant, practical information toward managing their illness [34-36]. Our survey also demonstrates that individuals are strongly motivated to share PHI if it positively impacts their children’s or others’ lives.

Similar to published literature [6,34], we found that attention to optimizing web privacy and security is critical when developing a new digital health platform. Establishing digital trust can be achieved via internal technological features as well as external validation of the technology by trusted sources. Internal security features include two-factor authentication and encrypted storage of PHI [37]. Our study also identified notification of account changes or activity, using a trusted sign-in partner, and strong minimum password requirements as important in gaining digital trust. External features that enhance digital trust include recommended use by trusted HCPs or health care organizations [38,39] or endorsements by friends, family, or other patients or caregivers. In addition, research by Graham et al [25] highlights the significance of collaboration between researchers and patients, underscoring the importance of co-design approaches. This collaborative effort enables teams to gain deeper insights into the needs of end users (eg, security features), facilitating the development of interventions that are more aligned with user preferences and expectations [22]. Through iterative co-design processes, there is a potential for enhanced user engagement and overall user experience, ensuring that the mobile applications effectively address its users’ needs.

### Strengths and Limitations

Our study had both limitations and strengths. First, our survey instrument was not validated and was a voluntary, self-report survey introducing the potential for information, recall, social desirability, and sampling bias. We used Insight West’s panel of volunteer participants to mitigate bias and maximize study validity by accessing a large sample size, yet we acknowledge our results should be interpreted with caution. For example, participants self-reported their chronic disease status and people who have inherent distrust in sharing information on the web might have been less likely to participate in our web-based survey. Despite using volunteer panels, our survey sample was overrepresented by participants living in urban or suburban areas and those with higher levels of education and household income. Furthermore, we could not stratify our findings by ethnicity or race or family structure (two-parent vs single-parent homes). Consequently, the validity of our survey results is challenged, potentially affecting the accuracy of estimations regarding relationships between variables and the generalizability of this study’s findings to the entire North American population. Second, the regression analysis was exploratory in nature with the possibility of residual confounding, and therefore, results should be interpreted with caution. Third, our sample size for the qualitative bulletin boards was small, limiting our qualitative analyses and ability to triangulate quantitative and qualitative data. As such, these findings cannot be viewed as representative and are only presented in this paper to supplement the quantitative survey findings. Finally, our survey data were gathered at the peak of the COVID-19 pandemic, a period marked by a surge in enthusiasm and adoption of digital health tools out of necessity. Therefore, it is essential not to overlook the potential confounding effect of the pandemic on attitudes toward these technologies at the time of data collection. Study strengths included a mixed methods design and a robust sample size of >1000 respondents from across Canada and the United States, strengthening the generalizability of our results. Further, we included the perspectives of youth aged 16 to 17 years on digital trust, which is understudied.

### Conclusions and Future Directions

Our research confirms that there is a willingness among caregivers and youth to use a digital platform like TrustSphere for clinical care delivery and to share their PHI for not-for-profit research. However, perceptions around digital trust vary across sociodemographic groups. Therefore, when designing digital applications, diverse engagement of end users is essential. The results of this study will inform the prioritization of the technological features of TrustSphere’s “digital front door” and have validated the importance of engaging end users (patients or caregivers and health care professionals) as early as possible in the iterative co-design of TrustSphere to optimize the value of the digital tool and ultimately enhance digital trust. Broadly, this study provides much-needed guidance to researchers and technology developers on what it takes to overcome the barrier of digital trust that has, to date, impeded the comprehensive uptake of digital health platforms. Additional research is needed to characterize the digital needs of underrepresented or vulnerable groups to ensure that digital health solutions are accessible to all.

## Appendix

Multimedia Appendix 1 TrustSphere adult survey.

Multimedia Appendix 2 TrustSphere youth survey.

Multimedia Appendix 3 Web-based discussion board question list.

Multimedia Appendix 4 Security processes and mechanisms that would improve trust in a digital platform.

Multimedia Appendix 5 Comfort levels in sharing your child’s personal health information.

## Abbreviations

CROSS: Consensus-Based Checklist for Reporting of Survey Studies
HCP: health care provider
OR: odds ratio
PHI: personal health information
SRQR: Standards for Reporting Qualitative Research
T1D: type 1 diabetes
