# Supplementary material for: Caregiver and Youth Characteristics That Influence Trust in Digital Health Platforms in Pediatric Care: Mixed Methods Study
Source: J Med Internet Res. 2024 Oct 28;26:e53657. doi: 10.2196/53657 (PMC11555442; doi:10.2196/53657)
Supplement: Multimedia Appendix 1 [file jmir_v26i1e53657_app1.docx]

**TRUSTSPHERE – Insights West Adult Panel Survey**

| INTRODUCTION |
| --- |

Thank you for taking the time to complete this survey. It should take approximately 12-15 minutes to complete.

S1. To start, please select the country you live in.

USA

Canada

Mexico **[THANK & TERMINATE]**

[ask only in canada]

S2. Which province or territory do you live in?

Choose one.

**[DO NOT RANDOMIZE]**

British Columbia

Alberta

Saskatchewan

Manitoba

Ontario

Quebec

Nova Scotia

New Brunswick

Prince Edward Island

Newfoundland

Yukon Territory

Northwest Territories

Nunavut

Outside Canada **[THANK & TERMINATE]**

[ask only in us]

S8. Which state do you live in?

Choose one.

**[DO NOT RANDOMIZE]**

Alabama [SOUTH]

Alaska [WEST]

Arizona [WEST]

Arkansas [SOUTH]

California [WEST]

Colorado [WEST]

Connecticut [NORTHEAST]

Delaware [SOUTH]

District of Columbia [SOUTH]

Florida [SOUTH]

Georgia [SOUTH]

Hawaii [WEST]

Idaho [WEST]

Illinois [MIDWEST]

Indiana [MIDWEST]

Iowa [MIDWEST]

Kansas [MIDWEST]

Kentucky [SOUTH]

Louisiana [SOUTH]

Maine [NORTHEAST]

Maryland [SOUTH]

Massachusetts [NORTHEAST]

Michigan [MIDWEST]

Minnesota [MIDWEST]

Mississippi [SOUTH]

Missouri [MIDWEST]

Montana [WEST]

Nebraska [MIDWEST]

Nevada [WEST]

New Hampshire [NORTHEAST]

New Jersey [NORTHEAST]

New Mexico [WEST]

New York [NORTHEAST]

North Carolina [SOUTH]

North Dakota [MIDWEST]

Ohio [MIDWEST]

Oklahoma [SOUTH]

Oregon [WEST]

Pennsylvania [NORTHEAST]

Rhode Island [NORTHEAST]

South Carolina [SOUTH]

South Dakota [MIDWEST]

Tennessee [SOUTH]

Texas [SOUTH]

Utah [WEST]

Vermont [NORTHEAST]

Virginia [SOUTH]

Washington [WEST]

West Virginia [SOUTH]

Wisconsin [MIDWEST]

Wyoming [WEST]

SCRIPTWRITER TO CREATE REGION VARIABLE FROM S8

Northeast

Midwest

South

West

S3. To start, do you have any children in the following age groups living in your household?

*Select all that apply.*

Less than 5 years

5-7 years

8-10 years

11-13 years

14-15 years

16-17 years

No children under 18 years **[EXCLUSIVE] [THANK & TERMINATE]**

S4. Which of the following best describes the role you play when it comes to making decisions about your child’s/children’s health?

*Please select one.*

I am the primary/sole decision maker

I share the decision-making responsibility with another parent/guardian

I have no role or influence in making decisions regarding my child’s/children’s health **[THANK & TERMINATE]**

**[IF HAVE CHILD AGE 8+]**

S4b. Which of the following best describes the role **your child/children** play when it comes to making decisions about their health?

*Please select one.*

They share the decision-making responsibility

They have no role or influence in making decisions

**[ASK OF ALL]**

S5. Do you, or does anyone in your household, work in a market research department or company?

Yes **[THANK AND TERMINATE]**

No

| A - Overall Trust |
| --- |

A1. In general, what is your level of concern regarding data privacy and security issues (e.g. personal data being hacked, or companies such as Google or Facebook tracking your activities) when you are engaging in online activity?

*Select one.*

**[COLUMNS]**

Extremely concerned

Very concerned

Somewhat concerned

Unconcerned

Very unconcerned

| Current Understanding of Data Storage, Transfer and Security |
| --- |

B1. To start, which of the following types of healthcare providers has **your child** **or children** visited either in person or virtually in the past year?

*Select all that apply.*

**[RANDOMIZE]**

Nutritionist or dietitian

Pediatrician

Massage therapist

Acupuncturist

Chiropractor

Psychologist or psychiatrist

Physical therapist or occupational therapist

Kinesiologist or rehabilitation specialist

Optometrist

Family Doctor

Nurse Practitioner

Doctor at drop-in clinic

Specialist doctor or nurse at hospital

Pharmacist

School nurse

Other (please specify) **[ANCHOR]**

None of the above **[EXCLUSIVE] [ANCHOR] > Skip to B3**

B2. How did your child/children visit with each of the healthcare providers?
*Select one answer per row.*

**[ROWS]**

**[INSERT ALL SELECTED @ B1]**

**[COLUMNS]**

In person

Virtually/online

Both

B3. Below are a series of statements related to your perception of security of data and personal information in healthcare.  Please rate how much you agree or disagree with each statement.

*Select one answer per row.*

**[ROWS – RANDOMIZE]**

I have a clear understanding of how and where my child’s/children’s health information is stored

I trust that my healthcare provider will keep my child’s/children’s health information secure

I trust that there are government regulations and practices in place to ensure health information is kept secure

I trust that there are organizational (e.g. hospital) regulations and practices in place to ensure health information is kept secure

I have a clear understanding of who can access my child’s/children’s health information

I’m willing to share some of my child’s/children’s health information confidentially if it helps create progress in non-profit health research

**[COLUMNS]**

Strongly disagree

Somewhat disagree

Somewhat agree

Strongly agree

B4. In what way(s) do you think the healthcare providers your child/children see store their personal health records?

*Select all that apply.*

Paper copies that are secured on-site

Secure computer system that can only be accessed on-site

Secure computer system that can be accessed online

B5. There are many types of healthcare providers that may collect or have access to your child’s health information. What is your understanding of how your child’s health information is shared between healthcare providers your child/children might visit?

*Select one.*

**Never shared** between the healthcare providers that my child sees

**Sometimes shared** between the other healthcare providers that my child sees

**Usually** shared between the other healthcare providers that my child sees

**Always shared between** the other healthcare providers that my child sees

B6. In which instances do you believe **explicit consent from a child’s parent/guardian is currently required** for a child’s health information to be shared?

*Select all that apply.*

**[DO NOT RANDOMIZE]**

If your healthcare provider shares your child’s health information **with another healthcare provider that your child sees**

If your healthcare provider shares your child’s confidential health information **with another healthcare provider that your child doesn’t see** (e.g. for a consultation or second opinion)

If your healthcare provider shares your child’s confidential health information **with a non-profit public research organization working to discover a new general medical treatment**

If your healthcare provider shares your child’s confidential health information **with a non-profit public research organization working to discover a new medical treatment for a condition your child has**

None of these **[EXCLUSIVE]**

B7a. For these examples in which you believe explicit consent is required, please indicate **for each instance how you feel about the consent requirement and whether you would be likely to give consent** for your child’s health information to be shared?

*Select one answer per row.*

**[ROWS]**

**[INSERT ALL SELECTED @ B6]**

**[COLUMNS]**

Consent should **not** be required

Consent **should** be required, and I **am** likely to consent

Consent **should** be required, and I am **not** likely to consent

B7b. For these examples in which you believe explicit consent is **not** required, please indicate **in which instances you think it is acceptable, and in which instances you think it is unacceptable,** that your child’s health information would be shared without your explicit consent?

*Select one answer per row.*

**[ROWS]**

**[INSERT ALL NOT SELECTED @ B6]**

**[COLUMNS]**

Acceptable

Unacceptable

B8. To what degree to do you trust yourself to evaluate the ethical considerations (such as privacy, risks and benefits) and give consent to use your child’s health information for non-profit public research?

*Select one answer per row.*

**[COLUMNS]**

A great deal

A moderate amount

A small amount

Not at all

B9. To what degree to do you trust the following types of health care professionals and institutions to evaluate the ethical considerations (such as privacy, risks and benefits) and give consent on your behalf to use your child’s health information for non-profit public research*?*

*Select one answer per row.*

**[ROWS]**

Your healthcare institution

Insurance providers

Healthcare providers

Health technology companies

Non-profit research institutions such as universities

**[COLUMNS]**

A great deal

A moderate amount

A small amount

Not at all

B10. Which of the following factors would **motivate** you to share your child’s health information for non-profit medical research?

*Rank your top 3.*

**[ROWS -RANDOMIZE]**

Being able to find out the outcome of the research

Knowing how the findings are being used

Remaining anonymous

Being able to do something positive to help others

Doing something that may have a positive impact on my child’s health

Working on an issue that is important to me and/or my child

Being able to see exactly who currently has access to the data

Being able to see exactly who has previously accessed the data

Being able to select the specific information that would be shared

Having a trusted healthcare provider verify the integrity and security of the research

Having a trusted group that includes ethics experts, patients, scientists and others verify the integrity and security of the research

B11. What do you believe are the most important pieces of your child’s health information that might be shared among healthcare providers to **improve the quality of care that your child receives**?

*Select your top 3.*

**[RANDOMIZE]**

Lab test results

Medications, procedures and treatments (past and present)

Diagnoses

Medical imaging (e.g. x-rays or ultrasounds)

A list of your child’s other healthcare providers

Health habits (physical activity; nutrition/eating; sleep habits; screen time)

Data from apps (e.g. nutrition, mental health, activity, etc)

Data from health devices (e.g. monitors, pumps, smart watches, step counters, etc)

Infant feeding (breastfeeding/formula feeding/introducing solids)

Mental/emotional health

Immunization records

Dental health

Allergies

Family medical history

Other – [specify] [ANCHOR]

None of these – no information about my child should be shared [EXCLUSIVE] [ANCHOR]

B12. What do you believe are the most important pieces of your child’s health information that can be shared among non-profit researchers and used in research **for public benefit**?

*Select your top 3.*

**[RANDOMIZE]**

INSERT SAME LIST AS PREVIOUS (B11)

None of these – no information about my child should be shared [EXCLUSIVE] [ANCHOR]

B13. Do you or your child wear/use any of the following types of health monitoring equipment?

*Select all that apply.*

**[ROWS - RANDOMIZE]**
Smart watches (e.g. Fitbit, Apple Watch)

Blood pressure monitoring

Continuous glucose monitoring system (CGMS)

Insulin Pump

Smart insulin pen

Health and wellbeing monitoring apps on smartphone

Do not use any these **[EXCLUSIVE]**

Other (please specify)

**[COLUMNS]**

Myself

My child

C1. For the next set of questions, we’d like you to review the description of a potential digital platform (for example, website or app) that would connect health care providers, and allow them and you to access your child’s health information.

*A secure online platform that will be customized for child and youth patients and their caregivers, and will integrate a patient’s health information such as diagnoses, medications and treatments, appointments, lab test results, wearable data (e.g. FitBit), etc. This platform would use secure and trusted digital identification, and follow the highest healthcare industry and public standards of privacy protection. The platform would help make it easier for children and families to access their health information and care plans, and to communicate directly with healthcare providers. It would also allow users to share their health information and care plans, if desired, with others involved in their child’s care, as well as donate their data confidentially for research.*

C2. How much do you agree or disagree with following statements about the concept you just read?

*Select one answer per row.*

**[ROWS - RANDOMIZE]**

A platform like this would be a positive change in how my child’s health information is stored

I would be concerned about the security of this platform holding everyone’s health information

I would share my child’s information on this platform with multiple providers

I would trust that myself and those I approve would be the only ones who could access my child’s health information

I would find it overwhelming to have to keep track of another important digital account

**[COLUMNS]**

Strongly disagree

Somewhat disagree

Somewhat agree

Strongly agree

C3. How helpful do you think an integrated platform as described above would be to/for the following groups and/or individuals?

*Select one answer per row.*

**ROWS**

Your child(ren)

You as a parent/guardian

Doctors and other healthcare practitioners

Researchers

**COLUMNS**

Not at all helpful

Not very helpful

Somewhat helpful

Very helpful

Extremely helpful

C4. Which of the following security processes or mechanisms would make you most trusting of this kind of digital platform?

*Rank your top 3.*

**[RANDOMIZE]**

Tap into phone’s security mechanisms (e.g. phone’s fingerprint scanner)

Multi-factor-authentication (e.g. a code sent to your phone when signing in)

Strong minimum password strength requirements

Using a trusted sign-in partner (e.g. signing in through your online banking, government services account, or existing healthcare patient portal that you use)

Being required to sign in again at regular time intervals

Notification of account changes and activity (including who has logged in and/or made changes)

Hosted on a URL (website name) that you recognize and/or trust

Other **[SPECIFY] [ANCHOR]**

Nothing/none of these [**EXCLUSIVE] [ANCHOR]**

I don’t know **[EXCLUSIVE] [ANCHOR]**

C5. If you had control over who could access the data, how comfortable would you be sharing the following types of your child’s health information on a platform like this?

*Select one answer per row.*

**[ROWS – RANDOMIZE]**

Demographic information (name, age, sex, date of birth, health card number, etc).

Your contact information (home address, phone number, email, etc.)

Lab test results

Diagnoses

Medications, procedures and treatments (past and present)

Medical imaging (e.g. x-rays or ultrasounds)

A list of your child’s other healthcare providers

Health habits (physical activity; nutrition/eating; sleep habits; screen time)

Data from apps (e.g. nutrition, mental health, activity, etc)

Data from health devices (e.g. monitors, pumps, smart watches, step counters, etc)

Infant feeding habits (breastfeeding/formula feeding/introducing solids)

Mental/emotional health

Immunization records

Family medical history

Dental health

Allergies

**[COLUMNS]**

Very uncomfortable

Somewhat uncomfortable

Somewhat comfortable

Very comfortable

C6. What is the likelihood that you would use a platform like this?

*Select one.*

Very unlikely

Unlikely

Undecided

Likely

Very likely

| Z. PROFIING (~x min) |
| --- |

The last few questions are for classification purposes only.

Z1. In what year were you born?

**[DROP DOWN RANGE 2004 to 1940]**

Prefer not to say

Z2. Do you or your child have a chronic disease that requires you access care regularly?

**[ROWS]**

Yourself

Your child

**[COLUMNS]**

Yes

No

Prefer not to answer

Z3. Do you have any of the following types of health care coverage?

*Choose all that apply.*

Private health care coverage or insurance that my employer pays for

Private health care coverage or insurance that I pay for

**[USA ONLY]** Public health care coverage

**[USA ONLY]** No health care coverage **[EXCLUSIVE]**

**[CANADA ONLY]** No additional health care coverage **[EXCLUSIVE]**

Z4. Which of the following genders do you identify as?

*Please select one.*

Male

Female

Other

Prefer not to answer

Z5. Which of the following best describes the area you live?

*Please select one.*

Urban

Suburban

Rural

Don’t know/prefer not to answer

Z6. Which of the following ranges best describes your **total annual household income** before taxes?

Please select one.

Less than $45,000

$45,000 to less than $75,000

$75,000 to less than $100,000

$100,000 to $150,000

$150,000 to $300,000

More than $300,000

Prefer not to answer

Z7. What is the highest level of education you have completed?

Choose one.

High school graduation or less

Some trade school

Graduated from trade school

Some college / university

University or college undergraduate degree, such as a bachelor’s degree

University or college graduate degree, such as a master’s or PhD

Prefer not to answer

X1. We are looking for parents who might be interested in participating in further discussion about this topic to help provide further direction on the healthcare platform.

The discussion will be held over 3 days in the coming months. You will be expected to log-in to the discussion on a private bulletin board for approximately 15-20 minutes on each of the 3 days to answer questions and respond to other people’s comments. You can participate at any time of the day, but we may ask that you come back and clarify some of your comments should it be required.

This discussion is strictly for exploratory purposes only, and we are only interested in hearing your opinions. You will remain completely anonymous to others in the discussion, and to reward you for taking the time to share your opinions, each participant will receive an incentive of **[$75** **CAD] [$50 USD]** upon completion of all three days.

Are you interested in being contacted about these groups when the dates have been determined?

Yes

No

**[IF HAVE TEEN 16-18 and LIVE IN CANADA]**

R1. As part of our research, we are looking to gain feedback from teens age 16-18 with regards to how they feel about their health. As their guardian, do you consent to being contacted with more information about having your teen participate in this research survey?

Yes

No

If yes, link to online parental consent form.

| CLOSING |
| --- |

[INFORMATION SCREEN]

Thank you very much for sharing your opinions with us; your feedback is greatly appreciated!
