# Supplementary material for: Caregiver and Youth Characteristics That Influence Trust in Digital Health Platforms in Pediatric Care: Mixed Methods Study
Source: J Med Internet Res. 2024 Oct 28;26:e53657. doi: 10.2196/53657 (PMC11555442; doi:10.2196/53657)
Supplement: Multimedia Appendix 2 [file jmir_v26i1e53657_app2.docx]

**TRUSTSPHERE – Insights West Youth Survey**

| INTRODUCTION |
| --- |

Thank you for taking the time to complete this survey. It should take approximately 10-12 minutes to complete.

S1. Which province or territory do you live in?

Choose one.

[DO NOT RANDOMIZE]

British Columbia

Alberta

Saskatchewan

Manitoba

Ontario

Quebec

Nova Scotia

New Brunswick

Prince Edward Island

Newfoundland

Yukon Territory

Northwest Territories

Nunavut

Outside Canada [THANK & TERMINATE]

S2. Which of the following best describes the role you play when it comes to making decisions about your own health?

*Please select one.*

I am the primary/sole decision maker

I share the decision-making responsibility with my parent(s)/guardian(s)

| A - Overall Trust |
| --- |

A1. In general, what is your level of concern regarding data privacy and security issues (e.g. personal data being hacked, or companies such as Google or Facebook tracking your activities) when you are engaging in online activity?

*Select one.*

**[COLUMNS]**

Extremely concerned

Very concerned

Somewhat concerned

Unconcerned

Very unconcerned

| Current Understanding of Data Storage, Transfer and Security |
| --- |

B1. To start, which of the following types of healthcare practitioners have you visited either in person or virtually in the past year?

*Select all that apply.*

**[RANDOMIZE]**

Nutritionist or dietitian

Pediatrician

Massage therapist

Acupuncturist

Chiropractor

Personal trainer

Psychologist or psychiatrist

Physical therapist or occupational therapist

Kinesiologist or rehabilitation specialist

Optometrist

Family Doctor

Nurse Practitioner

Doctor at drop-in clinic

Specialist doctor or nurse at hospital

Pharmacist

School nurse

Other (please specify) **[ANCHOR]**

None of the above **[EXCLUSIVE] [ANCHOR] > Skip to B3**

B2. How did you visit with each of the healthcare practitioners?
*Select one answer per row.*

**[ROWS]**

**[INSERT ALL SELECTED @ B1]**

**[COLUMNS]**

In person

Virtually/online

Both

B3. Below are a series of statements related to your perception of security of data and personal information in healthcare.  Please rate how much you agree or disagree with each statement.

*Select one answer per row.*

**[ROWS – RANDOMIZE]**

I have a clear understanding of how and where my health information is stored

I trust that my healthcare provider will keep my health information secure

I trust that there are government regulations and practices in place to ensure health information is kept secure

I trust that there are organizational (e.g. hospital) regulations and practices in place to ensure health information is kept secure

I have a clear understanding of who can access my health information

I’m willing to share some of my health information anonymously if it helps create progress in non-profit health research

**[COLUMNS]**

Strongly disagree

Somewhat disagree

Somewhat agree

Strongly agree

B4. In what way(s) do you think the healthcare providers you see store your personal health records?

*Select all that apply.*

Paper copies that are secured on-site

Secure computer database that can only be accessed on-site

Secure computer database that can be accessed online

B5. There are many types of healthcare providers that may collect or have access to your health information. What is your understanding of how your health information is shared between health care providers you might visit?

*Select one.*

**Never shared** between the healthcare providers that I see

**Sometimes shared** between the other healthcare providers that I see

**Always shared between** the other healthcare providers that I see

B6. In which instances do you believe **explicit consent from you is currently required** for your health information to be shared?

*Select all that apply.*

**[DO NOT RANDOMIZE]**

If my healthcare provider shares my health information with another healthcare provider that I see

If my healthcare provider shares my confidential health information with another healthcare provider that I don’t see (e.g. for a consultation or second opinion)

If my healthcare provider shares my confidential health information with non-profit research organization working to discover a new general medical treatment

If my healthcare provider shares my confidential health information with non-profit research organization working to discover a new medical treatment for a medical condition I have

None of these **[EXCLUSIVE]**

B7a. For these examples in which you believe explicit consent is required, please indicate **for each instance how you feel about the consent requirement and whether you would be likely to give consent** for your health information to be shared?

*Select one answer per row.*

**[ROWS]**

**[INSERT ALL SELECTED @ B6]**

**[COLUMNS]**

Consent should **not** be required

Consent **should** be required, and I **am** likely to consent

Consent **should** be required, and I am **not** likely to consent

B7b. For these examples in which you believe explicit consent is **not** required, please indicate **in which instances you think it is acceptable, and in which instances you think it is unacceptable,** that your health information would be shared without your explicit consent?

*Select one answer per row.*

**[ROWS]**

**[INSERT ALL NOT SELECTED @ B6]**

**[COLUMNS]**

Acceptable

Unacceptable

B8. To what degree to do you trust yourself to evaluate the ethical considerations (such as privacy, risks and benefits) and give consent to use your health information for non-profit public research?

*Select one answer per row.*

**[COLUMNS]**

A great deal

A moderate amount

A small amount

Not at all

B9. To what degree to do you trust the following types of health care professionals and institutions to evaluate the ethical considerations (such as privacy, risks and benefits) and give consent on your behalf to use your health information for non-profit public research*?*

*Select one answer per row.*

**[ROWS]**

Your healthcare institution

Insurance providers

Healthcare providers

Health technology companies

Non-profit research institutions such as universities

**[COLUMNS]**

A great deal

A moderate amount

A small amount

Not at all

B10. Which of the following factors would **motivate** you to share your health information for non-profit medical research?

*Rank your top 3.*

**[ROWS -RANDOMIZE]**

Being able to find out the outcome of the research

Knowing how the findings are being used

Remaining anonymous

Being able to do something positive to help others

Doing something that may have a positive impact on my health

Working on an issue that is important to me

Being able to see exactly who currently has access to the data

Being able to see exactly who has previously accessed the data

Being able to select the specific information that would be shared

Having a trusted health provider verify the ethics and security of the research

Having a trusted group that includes ethics experts, patients, scientists and others verify the integrity and security of the research

B11. What do you believe are the most important pieces of your health information that might be shared among healthcare providers to **improve the quality of care that you receive**?

*Select your top 3.*

**[RANDOMIZE]**

Lab test results

Medications, procedures and treatments (past and present)

Diagnoses

Medical imaging (e.g. x-rays or ultrasounds)

A list of my other healthcare providers

Health Habits (physical activity; nutrition/eating; sleep habits; screen time)

Data from apps (e.g. nutrition, mental health, activity, etc)

Data from health devices (e.g. monitors, pumps, smart watches, step counters, etc)

Mental/emotional health

Immunization records

Dental health

Allergies

Family medical history

Other – [specify] [ANCHOR]

None of these – no information about me should be shared [EXCLUSIVE] [ANCHOR]

B12. What do you believe are the most important pieces of your health information that can be shared among non-profit researchers and used in research **for public benefit**?

*Select your top 3.*

**[RANDOMIZE]**

INSERT SAME LIST AS PREVIOUS (B11)

None of these – no information about me should be shared [EXCLUSIVE] [ANCHOR]

B13. Do you wear/use any of the following types of health monitoring equipment?

*Select all that apply.*

**[ROWS - RANDOMIZE]**
Smart watches (e.g. Fitbit, Apple Watch)

Blood pressure monitoring

Continuous glucose monitoring system (CGMS)

Insulin Pump

Smart insulin pen

Health and wellbeing monitoring apps on smartphone

Do not use any these **[EXCLUSIVE]**

Other (please specify)

C1. For the next set of questions, we’d like you to review the description of a potential digital platform (for example, website or app) that would connect health care providers, and allow them and you to access your health information.

*A secure online platform that will be customized for child and youth patients and their caregivers, and will integrate a patient’s health information such as diagnoses, medications and treatments, appointments, lab test results, wearable data (e.g. FitBit), etc. This platform would use secure and trusted digital identification, and follow the highest healthcare industry and public standards of privacy protection. The platform would help make it easier for children, youth and families to access their health information and care plans, and to communicate directly with healthcare providers. It would also allow users to share their health information and care plans, if desired, with others involved in their care, as well as donate their data confidentially for research.*

C2. How much do you agree or disagree with following statements about the concept you just read?

*Select one answer per row.*

**[ROWS - RANDOMIZE]**

A platform like this would be a positive change in how my health information is stored

I would be concerned about the security of this platform holding everyone’s health information

I would share my information on this platform with multiple providers

I trust that myself and those I approve would be the only ones who could access my health information

I would find it overwhelming to have to keep track of another important digital account

**[COLUMNS]**

Strongly disagree

Somewhat disagree

Somewhat agree

Strongly agree

C3. How helpful do you think an integrated platform as described above would be to/for the following groups and/or individuals?

*Select one answer per row.*

**ROWS**

You

Your parent(s)/guardian(s)

Doctors and other healthcare practitioners

Researchers

**COLUMNS**

Not at all helpful

Not very helpful

Somewhat helpful

Very helpful

Extremely helpful

C4. Which of the following security processes or mechanisms would make you most trusting of this kind of digital platform?

*Rank your top 3.*

**[RANDOMIZE]**

Tap into phone’s security mechanisms (e.g. phone’s fingerprint scanner)

Multi-factor-authentication (e.g. a code sent to your phone when signing in)

Strong minimum password strength requirements

Using a trusted sign-in partner (e.g. signing in through your online banking, government services account, or existing healthcare patient portal that you use)

Being required to sign in again at regular time intervals

Notification of account changes and activity (including who has logged in and/or made changes)

Hosted on a URL (website name) that you recognize and/or trust

Other **[SPECIFY] [ANCHOR]**

Nothing/none of these [**EXCLUSIVE] [ANCHOR]**

I don’t know **[EXCLUSIVE] [ANCHOR]**

C5. If you had control over who cold access the data, how comfortable would you be sharing the following types of your health information on a platform like this?

*Select one answer per row.*

**[ROWS – RANDOMIZE]**

Demographic information (name, age, sex, date of birth, health card number, etc).

Contact information (home address, phone number, email, etc.)

Lab test results

Medications, procedures and treatments (past and present)

A list of your other healthcare providers

Health habits (physical activity; nutrition/eating; sleep habits; screen time)

Data from apps (e.g. nutrition, mental health, activity, etc)

Data from health devices (e.g. monitors, pumps, smart watches, step counters, etc)

Mental/emotional health

Immunization records

Family medical history

Dental health

Allergies

**[COLUMNS]**

Very uncomfortable

Somewhat uncomfortable

Somewhat comfortable

Very comfortable

C6. What is the likelihood that you would use a platform like this?

*Select one.*

Very unlikely

Unlikely

Undecided

Likely

Very likely

| Z. PROFIING (~x min) |
| --- |

The last few questions are for classification purposes only.

Z1. Do you have a chronic disease that requires you access care regularly?

**[COLUMNS]**

Yes

No

Prefer not to answer

Z2. Which of the following genders do you identify as?

*Please select one.*

Male

Female

Other

Prefer not to answer

Z3. Which of the following best describes the area you live?

*Please select one.*

Urban

Suburban

Rural

Don’t know/prefer not to answer

| CLOSING |
| --- |

[INFORMATION SCREEN]

Thank you very much for sharing your opinions with us; your feedback is greatly appreciated!
