# Supplementary material for: Caregiver and Youth Characteristics That Influence Trust in Digital Health Platforms in Pediatric Care: Mixed Methods Study
Source: J Med Internet Res. 2024 Oct 28;26:e53657. doi: 10.2196/53657 (PMC11555442; doi:10.2196/53657)
Supplement: Multimedia Appendix 3 [file jmir_v26i1e53657_app3.docx]

**Multimedia Appendix – Qualitative Discussion Board Questions**

1. What are your top concerns when sharing personal data and information online? How do you deal with these concerns?
2. How aligned do you feel you and your child(ren) are in terms of concern about **data security**?
3. If there are other adults who also play a role in making decisions about your child's/children's health, how aligned do you feel you are with them with regard to concern about data security? Do they have any other concerns that you are aware of?
4. What places, if any, do you feel your personal data is kept secure in an online environment? Why do you feel your data is secure there?
5. Following are some key terms that we'll be discussing over the next few days with regard to data security and privacy. However, to start, we'd like to know what your current understanding of these terms is. Please share what these terms mean to you. If you don't know, that's OK too.
   1. Shared data access
   2. Consent
   3. Assent
6. Following are definitions of these terms that we would like you to keep in mind throughout the rest of our discussion.

*CONSENT: Consent is the agreement given by an adult or surrogate decision-maker (such as a parent or guardian) to a set of terms. Consent must be informed; meaning that you understand possible and benefits. Also, people must not feel pressured to provide their consent. Consent may be removed at any time if you ever decide that you no longer wish to share data.*

*ASSENT: In some cases, children may not have the ability to consent, for example, if they are very young. In these cases, it is required that we get that child's assent in addition to the consent of their surrogate decision-maker (such as a parent or guardian). Assent means that we have a child's agreement to go ahead with what their parent or guardian consented to. Children can understand some aspects of data sharing and so their willingness to share their data must be respected and may even override the preferences of their parent or guardian.*

For each of the definitions provided, please share if there is anything that you didn't think of or learned from them.

1. After reading these definitions, do you feel you have a clear understanding of the difference between consent and assent? Is there anything missing in these definitions that would help you understand them more?
2. Is there anything in there that you feel is not necessary to include in these definitions?
3. We'd now like you to review the definition of shared data access below.

*SHARED DATA ACCESS: is when health data can be accessed by approved researchers, clinicians, or service providers. In some cases, your approval (consent), and the approval of your child is required for this data to be shared.*

Please share if there is anything that you didn't think of, or learned from this definition of shared data access?

1. Is there anything that is still unclear in this definition?
2. What concerns, if any, do you have regarding shared health data access?
3. What information is most important for you to see or know before feeling comfortable giving consent to share your child's health data?
4. How much would you involve your child in your health care decisions to provide assent and consent to sharing health data? How or when do you think you will know if your child is ready to be involved in these decisions?
5. To start, what people, organizations or types of institutions would you be most comfortable giving consent to share your child's health data with, and for what purposes? For any listed, please explain why. If there are none, please also say why this is.
6. Have you ever been approached to participate in a university or hospital research study which required you to share your child's health data?

If you have been approached, what was the outcome of this request - did you agree to participate? Why or why not?

If you have not been approached, how do you think you would respond to the request?

1. What do you think children's health data that is shared with university or hospital researchers will be used for?
2. What concerns, if any, do you have about sharing your child's health data with university or hospital researchers? Why do you have these concerns?
3. What would make you more comfortable or trusting of sharing your child's health data with university or hospital researchers? What are the questions you would need to be addressed, or information you would need to be provided?
4. How, if at all, might the following pieces of information impact your decision to share your child's health data with university or hospital researchers? We know that there are several items listed below, please respond to each, unless you feel you your answer applies to more than one.
   1. They data sharing process is vetted by a trusted source like your doctor
   2. They are doing research that directly benefits your child
   3. The whole data sharing process is governed by a group that includes parents like yourself
   4. The information is stripped of information that could identify your child
   5. Your child will be anonymous (information will be stripped of any identifiable features) may be a better way of phrasing this
   6. You will be notified each time a study accesses your data
   7. The data may support new technologies and innovations
5. To start our last day of discussion, if you/your child were diagnosed with a chronic health condition, would you seek digital services like apps? Would you do this before or after you see a clinician? Where would you look for these apps?
6. For the next set of questions, we'd like you to review the description of a potential digital platform (for example, website or app) that would connect health care providers and allow them, and you, to access your child's health information.

*A secure online platform that will be customized for child and youth patients and their caregivers, and will integrate a patient's health information such as diagnoses, medications and treatments, appointments, lab test results, wearable data (e.g. FitBit), etc. This platform would use secure and trusted digital identification, and follow the highest healthcare industry and public standards of privacy protection. The platform would help make it easier for children and families to access their health information and care plans, and to communicate directly with healthcare providers. It would also allow users to share their health information and care plans, if desired, with others involved in their child's care, as well as donate their data confidentially for research*.

After reading this description, is there anything that is unclear to you? What questions do you have that would need to be addressed before you would consider providing consent to share your child's personal health data on it?

1. If this app was recommended by your physician would you use it? Why or why not?
2. If this app was not recommended by your physician, what might motivate you to seek it out?
