# Supplementary material for: Caregiver and Youth Characteristics That Influence Trust in Digital Health Platforms in Pediatric Care: Mixed Methods Study
Source: J Med Internet Res. 2024 Oct 28;26:e53657. doi: 10.2196/53657 (PMC11555442; doi:10.2196/53657)
Supplement: Multimedia Appendix 4 [file jmir_v26i1e53657_app4.docx]

**Table S1:** Security processes/mechanisms that would improve trust in a digital platform. (n=1301)

|  | **Adults and Youth** | | **Parent Has Chronic Disease** | | **Child Has Chronic Disease** | | **Annual Household Income** | | |
| --- | --- | --- | --- | --- | --- | --- | --- | --- | --- |
|  | Adults (N = 1128) | Youth (N = 173) | Yes (N = 231) | No (N=766) | Yes (N = 198) | No  (N = 873) | Less than $75,000 (N = 326) | $75,000 to $150,000  (N = 529) | More than $150,000 (N = 216) |
|  | N (%) | N (%) | N (%) | N (%) | N (%) | N (%) | N (%) | N (%) | N (%) |
| **Which of the following security processes or mechanisms would make you most trusting of this kind of digital platform? Rank your top 3.** | | | | | | | | | |
| Being required to sign in again at regular time intervals | 285 (32.2) | 54 (35.8) | 79 (39.3) | 185 (31.7) | 50 (28.4) | 219 (32.5) | 71 (30.2) | 146 (34.4) | 59 (32.1) |
| Hosted on a URL (website name) that you recognize and/or trust | 224 (25.3) | 42 (27.8) | 61 (30.3) | 138 (23.7) | 48 (27.3) | 165 (24.5) | 62 (26.4) | 110 (25.9) | 43 (23.4) |
| Multi-factor-authentication (e.g. a code sent to your phone when signing in) | 539 (60.8) | 85 (56.3) | 109 (54.2) | 363 (62.3) | 109 (61.9) | 413 (61.4) | 144 (61.3) | 245 (57.6) | 124 (67.4) |
| Notification of account changes and activity (including who has logged in and/or made changes) | 504 (56.9) | 74 (49.0) | 101 (50.2) | 328 (56.3) | 108 (61.4) | 374 (55.6) | 131 (55.7) | 242 (56.9) | 104 (56.5) |
| Strong minimum password strength requirements | 412 (46.5) | 76 (50.3) | 88 (43.8) | 287 (49.2) | 70 (39.8) | 326 (48.4) | 116 (49.4) | 194 (45.6) | 81 (44.0) |
| Tap into phone’s security mechanisms (e.g. phone’s fingerprint scanner) | 247 (27.9) | 46 (30.5) | 69 (34.3) | 149 (25.6) | 57 (32.4) | 178 (26.4) | 90 (38.3) | 127 (29.9) | 47 (25.5) |
| Using a trusted sign-in partner (e.g. signing in through your online banking, government services account, or existing healthcare patient portal that you use) | 414 (46.7) | 68 (45.0) | 91 (45.3) | 275 (47.2) | 81 (46.0) | 319 (47.4) | 125 (53.2) | 194 (45.6) | 87 (47.3) |
| Other | 6 (0.7) | 0 (0) | 1 (0.5) | 4 (0.7) | 1 (0.6) | 4 (0.6) | 4 (1.7) | 1 (0.2) | 1 (0.5) |
| Number of complete & partial responses | 886 | 151 | 201 | 583 | 176 | 673 | 235 | 425 | 184 |
| Number of no responses | 242 | 22 | 30 | 183 | 22 | 200 | 91 | 104 | 32 |
