# Supplementary material for: Caregiver and Youth Characteristics That Influence Trust in Digital Health Platforms in Pediatric Care: Mixed Methods Study
Source: J Med Internet Res. 2024 Oct 28;26:e53657. doi: 10.2196/53657 (PMC11555442; doi:10.2196/53657)
Supplement: Multimedia Appendix 5 [file jmir_v26i1e53657_app5.docx]

**Table S2:** Comfort levels in sharing your child’s personal health information. (n=1301)

|  | | **Adults and Youth** | | **Parent Has Chronic Disease** | | **Child Has Chronic Disease** | | **Annual Household Income** | | |
| --- | --- | --- | --- | --- | --- | --- | --- | --- | --- | --- |
|  |  | Adults (N = 1128) | Youth (N = 173) | Yes (N = 231) | No (N=766) | Yes (N = 198) | No  (N = 873) | Less than $75,000 (N = 326) | $75,000 to $150,000  (N = 529) | More than $150,000 (N = 216) |
|  |  | N (%) | N (%) | N (%) | N (%) | N (%) | N (%) | N (%) | N (%) | N (%) |
| **If you had control over who could access the data, how comfortable would you be sharing the following types of your child’s health information on a platform like this?** | | | | | | | | | | |
| Demographics | Uncomfortable | 425 (37.6) | 48 (27.7) | 68 (29.4) | 319 (41.6) | 45 (22.7) | 354 (40.5) | 151 (46.3) | 172 (32.5) | 73 (33.7) |
|  | Comfortable | 694 (61.5) | 124 (71.6) | 163 (70.5) | 443 (57.8) | 149 (75.2) | 516 (59.1) | 174 (53.3) | 353 (66.7) | 143 (66.2) |
|  | No Response | 9 (0.7) | 1 (0.5) | 0 (0) | 4 (0.5) | 4 (2) | 3 (0.3) | 1 (0.3) | 4 (0.7) | 0 (0) |
| Contact Information | Uncomfortable | 479 (42.4) | 60 (34.6) | 77 (33.3) | 360 (46.9) | 52 (26.2) | 400 (45.8) | 161 (49.3) | 197 (37.2) | 91 (42.1) |
|  | Comfortable | 639 (56.6) | 111 (64.1) | 154 (66.6) | 402 (52.4) | 141 (71.2) | 470 (53.8) | 164 (50.3) | 328 (62) | 124 (57.4) |
|  | No Response | 10 (0.8) | 2 (1.1) | 0 (0) | 4 (0.5) | 5 (2.5) | 3 (0.3) | 1 (0.3) | 4 (0.7) | 1 (0.4) |
| Lab Test Results | Uncomfortable | 343 (30.4) | 40 (23.1) | 51 (22) | 272 (35.5) | 23 (11.6) | 301 (34.4) | 122 (37.4) | 131 (24.7) | 65 (30) |
|  | Comfortable | 770 (68.2) | 130 (75.1) | 179 (77.4) | 485 (63.3) | 171 (86.3) | 564 (64.6) | 200 (61.3) | 393 (74.2) | 149 (68.9) |
|  | No Response | 15 (1.3) | 3 (1.7) | 1 (0.4) | 9 (1.1) | 4 (2) | 8 (0.9) | 4 (1.2) | 5 (0.9) | 2 (0.9) |
| Diagnoses | Uncomfortable | 353 (31.2) | n/a | 59 (25.5) | 274 (35.7) | 27 (13.6) | 303 (34.7) | 122 (37.4) | 143 (27) | 63 (29.1) |
|  | Comfortable | 758 (67.1) | n/a | 172 (74.4) | 483 (63) | 166 (83.8) | 561 (64.2) | 201 (61.6) | 380 (71.8) | 149 (68.9) |
|  | No Response | 17 (1.5) | n/a | 0 (0) | 9 (1.1) | 5 (2.5) | 9 (1) | 3 (0.9) | 6 (1.1) | 4 (1.8) |
| Medications & Medical History | Uncomfortable | 336 (29.7) | 36 (20.8) | 55 (23.8) | 263 (34.3) | 19 (9.5) | 295 (33.7) | 107 (32.8) | 138 (26) | 67 (31) |
|  | Comfortable | 780 (69.1) | 135 (78) | 175 (75.7) | 497 (64.8) | 176 (88.8) | 571 (65.4) | 217 (66.5) | 385 (72.7) | 149 (68.9) |
|  | No Response | 12 (1) | 2 (1.1) | 1 (0.4) | 6 (0.7) | 3 (1.5) | 7 (0.8) | 2 (0.6) | 6 (1.1) | 0 (0) |
| Medical Imaging | Uncomfortable | 337 (29.8) | n/a | 49 (21.2) | 267 (34.8) | 23 (11.6) | 292 (33.4) | 121 (37.1) | 134 (25.3) | 58 (26.8) |
|  | Comfortable | 777 (68.8) | n/a | 181 (78.3) | 492 (64.2) | 169 (85.3) | 575 (65.8) | 203 (62.2) | 389 (73.5) | 156 (72.2) |
|  | No Response | 14 (1.2) | n/a | 1 (0.4) | 7 (0.9) | 6 (3) | 6 (0.6) | 2 (0.6) | 6 (1.1) | 2 (0.9) |
| List of HCPs | Uncomfortable | 331 (29.3) | 37 (21.3) | 50 (21.6) | 259 (33.8) | 23 (11.6) | 286 (32.7) | 107 (32.8) | 136 (25.7) | 64 (29.6) |
|  | Comfortable | 787 (69.7) | 133 (76.8) | 181 (78.3) | 502 (65.5) | 171 (86.3) | 583 (66.7) | 217 (66.5) | 389 (73.5) | 152 (70.3) |
|  | No Response | 10 (0.8) | 3 (1.7) | 0 (0) | 5 (0.6) | 4 (2) | 4 (0.4) | 2 (0.6) | 4 (0.7) | 0 (0) |
| Health Habits | Uncomfortable | 331 (29.3) | 41 (23.6) | 53 (22.9) | 255 (33.2) | 29 (14.6) | 285 (32.6) | 120 (36.8) | 132 (24.9) | 58 (26.8) |
|  | Comfortable | 788 (69.8) | 129 (74.5) | 178 (77) | 507 (66.1) | 165 (83.3) | 585 (67) | 204 (62.5) | 394 (74.4) | 158 (73.1) |
|  | No Response | 9 (0.7) | 3 (1.7) | 0 (0) | 4 (0.5) | 4 (2) | 3 (0.3) | 2 (0.6) | 3 (0.5) | 0 (0) |
| App Data | Uncomfortable | 366 (32.4) | 38 (21.9) | 55 (23.8) | 286 (37.3) | 32 (16.1) | 308 (35.2) | 125 (38.3) | 153 (28.9) | 63 (29.1) |
|  | Comfortable | 751 (66.5) | 133 (76.8) | 176 (76.1) | 474 (61.8) | 161 (81.3) | 560 (64.1) | 197 (60.4) | 373 (70.5) | 152 (70.3) |
|  | No Response | 11 (0.9) | 2 (1.1) | 0 (0) | 6 (0.7) | 5 (2.5) | 5 (0.5) | 4 (1.2) | 3 (0.5) | 1 (0.4) |
| Data from Health Devices | Uncomfortable | 362 (32) | 46 (26.5) | 55 (23.8) | 284 (37) | 25 (12.6) | 311 (35.6) | 122 (37.4) | 152 (28.7) | 68 (31.4) |
|  | Comfortable | 759 (67.2) | 126 (72.8) | 176 (76.1) | 480 (62.6) | 170 (85.8) | 560 (64.1) | 203 (62.2) | 375 (70.8) | 148 (68.5) |
|  | No Response | 7 (0.6) | 1 (0.5) | 0 (0) | 2 (0.2) | 3 (1.5) | 2 (0.2) | 1 (0.3) | 2 (0.3) | 0 (0) |
| Infant Feeding Habits | Uncomfortable | 346 (30.6) | n/a | 61 (26.4) | 265 (34.5) | 27 (13.6) | 292 (33.4) | 124 (38) | 136 (25.7) | 63 (29.1) |
|  | Comfortable | 760 (67.3) | n/a | 168 (72.7) | 493 (64.3) | 159 (80.3) | 573 (65.6) | 198 (60.7) | 386 (72.9) | 147 (68) |
|  | No Response | 22 (1.9) | n/a | 2 (0.8) | 8 (1) | 12 (6) | 8 (0.9) | 4 (1.2) | 7 (1.3) | 6 (2.7) |
| Mental/Emotional Health | Uncomfortable | 436 (38.6) | 54 (31.2) | 72 (31.1) | 328 (42.8) | 43 (21.7) | 364 (41.6) | 135 (41.4) | 181 (34.2) | 91 (42.1) |
|  | Comfortable | 677 (60) | 116 (67) | 158 (68.3) | 429 (56) | 152 (76.7) | 499 (57.1) | 188 (57.6) | 343 (64.8) | 122 (56.4) |
|  | No Response | 15 (1.3) | 3 (1.7) | 1 (0.4) | 9 (1.1) | 3 (1.5) | 10 (1.1) | 3 (0.9) | 5 (0.9) | 3 (1.3) |
| Immunization Records | Uncomfortable | 280 (24.8) | 30 (17.3) | 54 (23.3) | 205 (26.7) | 25 (12.6) | 235 (26.9) | 100 (30.6) | 103 (19.4) | 58 (26.8) |
|  | Comfortable | 835 (74) | 140 (80.9) | 176 (76.1) | 554 (72.3) | 169 (85.3) | 631 (72.2) | 224 (68.7) | 421 (79.5) | 156 (72.2) |
|  | No Response | 13 (1.1) | 3 (1.7) | 1 (0.4) | 7 (0.9) | 4 (2) | 7 (0.8) | 2 (0.6) | 5 (0.9) | 2 (0.9) |
| Family Medical History | Uncomfortable | 366 (32.4) | 42 (24.2) | 55 (23.8) | 285 (37.2) | 28 (14.1) | 311 (35.6) | 120 (36.8) | 143 (27) | 75 (34.7) |
|  | Comfortable | 746 (66.1) | 128 (73.9) | 175 (75.7) | 471 (61.4) | 166 (83.8) | 553 (63.3) | 202 (61.9) | 380 (71.8) | 139 (64.3) |
|  | No Response | 16 (1.4) | 3 (1.7) | 1 (0.4) | 10 (1.3) | 4 (2) | 9 (1) | 4 (1.2) | 6 (1.1) | 2 (0.9) |
| Dental Health | Uncomfortable | 271 (24) | 33 (19) | 41 (17.7) | 214 (27.9) | 17 (8.5) | 237 (27.1) | 99 (30.3) | 109 (20.6) | 44 (20.3) |
|  | Comfortable | 840 (74.4) | 137 (79.1) | 188 (81.3) | 543 (70.8) | 175 (88.3) | 628 (71.9) | 224 (68.7) | 414 (78.2) | 169 (78.2) |
|  | No Response | 17 (1.5) | 3 (1.7) | 2 (0.8) | 9 (1.1) | 6 (3) | 8 (0.9) | 3 (0.9) | 6 (1.1) | 3 (1.3) |
| Allergies | Uncomfortable | 275 (24.3) | 30 (17.3) | 51 (22) | 207 (27) | 16 (8) | 240 (27.4) | 102 (31.2) | 108 (20.4) | 48 (22.2) |
|  | Comfortable | 838 (74.2) | 141 (81.5) | 178 (77) | 553 (72.1) | 174 (87.8) | 629 (72) | 222 (68) | 414 (78.2) | 167 (77.3) |
|  | No Response | 15 (1.3) | 2 (1.1) | 2 (0.8) | 6 (0.7) | 8 (4) | 4 (0.4) | 2 (0.6) | 7 (1.3) | 1 (0.4) |
